# The Challenges of Projecting the Public Health Impacts of Marijuana Legalization in Canada: Comment on "Legalizing and Regulating Marijuana in Canada: Review of Potential Economic, Social, and Health Impacts"

**DOI:** 10.15171/ijhpm.2016.124

**Published:** 2016-09-10

**Authors:** Stephanie Lake, Thomas Kerr

**Affiliations:** ^1^British Columbia Centre for Excellence in HIV/AIDS, St. Paul’s Hospital, Vancouver, BC, Canada.; ^2^School of Population and Public Health, University of British Columbia, Vancouver, BC, Canada.; ^3^Department of Medicine, University of British Columbia, St. Paul’s Hospital, Vancouver, BC, Canada.

**Keywords:** Marijuana Legalization, Health Impact, Public Health, Canada

## Abstract

A recent editorial in this journal provides a summary of key economic, social, and public health considerations of the forthcoming legislation to legalize, regulate, and restrict access to marijuana in Canada. As our government plans to implement an evidence-based public health framework for marijuana legalization, we reflect and expand on recent discussions of the public health implications of marijuana legalization, and offer additional points of consideration. We select two commonly cited public concerns of marijuana legalization – adolescent usage and impaired driving – and discuss how the underdeveloped and equivocal body of scientific literature surrounding these issues limits the ability to predict the effects of legalization. Finally, we discuss the potential for some potential public health benefits of marijuana legalization – specifically the potential for marijuana to be used as a substitute to opioids and other risky substance use – that have to date not received adequate attention.

In light of the Canadian government’s plan to legalize, regulate, and restrict access to marijuana by Spring 2017, Dr. Hajizadeh’s editorial in this journal (*“Legalizing and regulating marijuana in Canada: review of potential economic, social, and health impacts”*)^1^ outlines a range of important points of consideration surrounding the potential economic, social, and public health impacts of marijuana legalization on a national level. Understandably, public health and safety are top concerns associated with the impending policy reform among the Canadian public. However, following a decade of Conservative government messaging that relied on exaggerated claims about the drug’s adverse health effects to reinforce its illegal status, discussions of the potential public health impacts of marijuana too often focus narrowly on a small set of findings that do not accurately reflect the state of the literature. As the newly elected Liberal government has expressed its intent to design an evidence-based framework that prioritizes public health, our aim is to review and expand upon points made in Dr. Hajizadeh’s editorial.

Sadly, at present, the ability to make informed projections about the health impacts of marijuana legalization has long been complicated by a range of factors, including incomplete scientific experimentation and observation resulting from the drug’s longstanding illegal status, and a complete lack of other nationwide legalization experiences. For example, though the effect of marijuana on youth is rarely overlooked in discussions of the potential public health impacts of legalization, whether legalization will actually pose a problem to Canada’s youth remains unknown. While there is some evidence linking marijuana use in adolescence to cognitive and mental health problems, including exacerbating schizophrenia in affected individuals,^2-4^ it is important to stress that the state of the literature in this area remains contested, as emerging research continues to challenge conclusions made from previous well-known studies that have concluded negative impacts of marijuana on the brain. For instance, we are now learning that previously established links between marijuana use and schizophrenia may simply reflect increased marijuana use among individuals with a pre-disposition to schizophrenia^5^; links between marijuana use and impaired neurodevelopment may reflect structural brain differences that preceded marijuana initiation^6^; and links between marijuana use and lower intelligence may reflect lower socio-economic status among marijuana users,^7^ cigarette and alcohol use,^8^ and/or differences in genetic make-up and environmental exposures throughout childhood and adolescence.^9^ However, while studies showing little-to-no impact of cannabis on adolescent brain development and functioning receive virtually no attention from mainstream media or government-funded research organizations (eg, the Canadian Centre for Substance Abuse),^10^ studies suggesting a negative impact are broadly cited with little critical evaluation.

Further, findings from studies of *frequent* marijuana use among youth tends to command the public discourse surrounding this topic, yet much less is known about the effect of *moderate* marijuana use among youth, which is an essential component to our understanding of the effect of marijuana on young people. Currently, roughly half of Canada’s youth report that marijuana is easily accessible,^11^ reflecting the highest rate of adolescent marijuana use of any developed nation.^12^ Considering the already high level of availability under the current system, many health and policy experts have long speculated that legalization is a favourable alternative to the current approach for preventing use among youth.^13,14^ This consensus is what largely justified the Liberal Government’s campaign promise to enact the legislation.^15^ In fact, based on the US medical marijuana experience, there is growing evidence to suggest that liberalization of marijuana policy does not promote increased usage among youth.^16,17^ Adolescent marijuana use, mental health, and cognitive development are often cited as concerns of legalization, but these points also warrant serious consideration of potential positive impacts of legalization – consideration that is most often ignored in media discourse.

Given the known impact of marijuana consumption on reduced attention span, motor skills, and slowed reaction time,^18^ motor vehicle injuries and fatalities are also a top public health concern of marijuana legalization. While several studies have examined the association between marijuana use and motor vehicle collisions, there is a high level of heterogeneity with regard to study design, study quality, and findings.^19^ A recent meta-analysis of the data estimates the risk to be significant yet low-to-moderate, and notes that the association is further reduced (yet still significant) after excluding studies that did not control for concurrent alcohol impairment.^19^ Determining whether marijuana legalization promotes increased motor vehicle collisions by way of increased prevalence of marijuana-impaired driving will undoubtedly be met with a host of challenges.

Dr. Hajizadeh cautions that Canada may see an increase in marijuana-related traffic deaths after legalization, similar to the apparent experience in Colorado. It is important to note that attributing any increase in marijuana-related traffic fatalities in Colorado to marijuana legalization itself is challenging if not impossible. Unlike alcohol, there is no consensus on a standard of “impairment” for tetrahydrocannabinol (THC) – the primary psychoactive compound in marijuana – and THC metabolites can be detected in the blood or urine several days after consuming marijuana.^20^ Thus an increase in the presence of THC among drivers who suffered fatalities in Colorado may have simply reflected an increase in marijuana use among adults who drive, rather than an increase in marijuana-impaired drivers.^21^ This is particularly plausible considering that the overall rate of fatal motor vehicle accidents in Colorado had been declining ahead of legalization^21^ and has continued to decline post legalization,^22^ despite an increase in the proportion of fatally injured drivers who test positive for THC. Finally, the possibility that legalization may promote increased marijuana testing among drivers needs to also be considered in these discussions. Drugged driving may prove to be a serious and legitimate concern of marijuana legalization; however, the current methods of determining marijuana impairment provide a clear impediment to evaluating the effect of legalization on motor vehicle accidents and fatalities. Overcoming this logistical challenge, which has yet to be publicly resolved by the Canadian government, will be critical to successful public health evaluation in this important area.

Finally, Dr. Hajizadeh discusses a range of potential perceived adverse impacts of marijuana legalization, but we want to point out that there may in fact be a range of potential positive outcomes that could be of interest in preparation for the legislation and its eventual evaluation. Although Dr. Hajizadeh acknowledges marijuana’s therapeutic benefits for certain health conditions including neuropathic pain, inflammatory bowel disease, and managing symptoms of chemotherapy and treatment-resistant epilepsy, these issues may be more relevant to Canada’s medical marijuana laws and physician prescribing guidelines rather than discussions of full-scale legalization. While marijuana is associated with certain adverse effects discussed both in Dr. Hajizadeh’s editorial and here, there is also a great deal of consensus within the scientific community that cannabis is comparatively safer than a host of other commonly abused substances including alcohol, methamphetamine, cocaine, and illicit opioids.^23^ A recent study using data from all 50 US states found a 25% reduction in opioid-related overdose deaths in states that enacted medical marijuana laws during the 10-year study period, relative to those that did not.^24^ While it is unclear if increased substitution of opioids with marijuana is driving this finding, research among users of medical marijuana from various regions across North America demonstrates that marijuana is often used as a substitute for other risky substance use.^25-27^ Canada’s soaring opioid dependence and overdose rates reflect a national public health crisis that requires a national and multi-faceted approach. Whether marijuana legalization will offer a sort of “harm reduction” component to Canada’s response to the opioid crisis in not known, but it should not be overlooked.

Canada is entering uncharted territory, as marijuana legalization of this magnitude is virtually void of scientific exploration. Furthermore, the body of research on the effects of marijuana alone remains underdeveloped, in large part due to its longstanding status as an illegal substance. Undoubtedly, Canada’s marijuana legalization will set a precedent for other governments looking to similarly reform marijuana legislation. We must ensure the policy is based on the science that has been presented fairly within the context of all evidence. Collectively, the single most important move that public health researchers and policy-makers can make is to put forward a long-term, adaptable research agenda that addresses a wide range of potential direct and indirect public health risks and benefits. A failure to do so will simply perpetuate the sad state of cannabis research internationally and result in policy that is not rooted in the best available evidence.

## Acknowledgments

We would to thank Tricia Collingham and Deborah Graham for their administrative assistance.

## Ethical issues

Not applicable.

## Competing interests

Authors declare that they have no competing interests.

## Authors’ contributions

TK and SL conceptualized the paper; SL wrote the initial manuscript draft; TK revised and contributed to subsequent drafts of the manuscript; both authors approved the final draft for submission.

## Authors’ affiliations

^1^British Columbia Centre for Excellence in HIV/AIDS, St. Paul’s Hospital, Vancouver, BC, Canada. ^2^School of Population and Public Health, University of British Columbia, Vancouver, BC, Canada. ^3^Department of Medicine, University of British Columbia, St. Paul’s Hospital, Vancouver, BC, Canada.
